# Southern Dental Association

**Published:** 1885-06

**Authors:** 


### Reports of Society Meetings.

### SOUTHERN DENTAL ASSOCIATION.

SEVENTEENTH ANNUAL MEETING, AT NEW ORLEANS.

Reported for the Independent Practitioner, by Mrs. M. W. J.

(Continued from page 265.)

SECOND DAY—MORNING SESSION.

Convened with B. H. Catching, Third Vice-President, in the chair.

The death of Dr. S. M. Prothro, of Chattanooga, was announced, and a committee appointed to frame memorial resolutions.

Dr. W. N. Morrison read a paper on Operative Dentistry, his subject being the too frequent reckless sacrifice of tooth material in opening dead teeth, for convenience of operating. Being liable to disintegration, they require the strongest walls. For root filling he uses very soft, pure gold wire, the exact size of the natural canal, never drilling or enlarging the latter. He fills the interstices with oxy-phosphate of zinc, or gutta percha, introduced with a tampon of bibulous paper to absorb the surplus fluid. He removes the contents of the canals and the pulp-chamber through a round aperture in the center of the crown, one-sixteenth of an inch in diameter. He described at length a tooth having a nodule in the pulp-chamber, too large to pass through the aperture. He rolled the nodule from side to side while extracting the pulp tissues and filling the roots, finally impacting it in the filling of the pulp-chamber. He devitalized the pulp with arsenic, removed, disinfected, and filled at one sitting.

A spirited discussion followed. Drs. Knapp and Morgan pronounced Dr. Morrison's position extreme, though assenting to the general principles laid down. Dr. Knapp would rather drill and operate through a mesial filling, than drill through the center of a sound crown. Dr. Morgan would prefer a slot, however narrow, to a round opening. He believed that immediately after the removal of a dead pulp was the worst time and worst condition for filling. The contents of the tubuli would decompose, the tooth would discolor, and mephitic gases might induce an abscess. He would cut away the dentine freely, because of the decomposition of the contents of the tubuli. He uses wire for root fillings, but with screw threads. Has found a case in which a root had been filled with oxy-chloride of zinc, and a ball of the material had been forced through the foramen.

*Dr. Parmly Brown*—Would not advise every one to try Dr. Morrison's opening. Where one would succeed ten would fail. He agreed with him as to immediate filling. He had successfully removed pulps, disinfected and filled, at one sitting, ten anterior superior teeth, in which pulps had been devitalized but not removed, six months previous; the fillings were all loose, and pus was exuding.

*Dr. Spalding*—Said such treatment would not do for the teeth of young children, but as a general rule he would insert the fillings immediately, unless the tooth was very sore. In every case he would fill the channels as quickly as possible. External conditions, even an outside abscess, were no hindrance, for he would treat the apex from the outside. If the periosteum is inflamed it is because of mephitic gases in the canal. If that be cleaned and filled the cause is removed. The canal must be perfectly dry to prevent chemical changes. He uses wire and gutta percha in the canals; gets the exact measure of depth of the canal and cuts the wire off; then pumps the canal full of gutta percha and insets the wire to the same depth it went before. He thus becomes certain that it is well filled, in breadth and depth.

*Dr. Harlan*—If the pulp is destroyed to-day, according to the laws governing physiology, changes from physiological to pathological conditions will occur. Nature requires eight days to remove eschar tissue. Mixed fluids entering will produce putrefaction. He extracts moisture, not with carbolic acid, not with creosote, but with absolute alcohol, which will not coagulate the tissues. It is not possible to perfectly fill curved roots with gold. We must use plastics to reach unequal surfaces. Oxy-phosphate and oxy-chloride of zinc harden before reaching the apex. He uses gutta percha, not heated in the flame or on a dish, not in cones, but drawn out fine, and sufficiently rigid to go to the apex.

*Dr. Williams*—Removes all tissues, disinfects thoroughly, and closes the apex with a lead cone, similar to the point of a lead pencil.

*Dr. Richie*—Is opposed to the capping of pulps, as an imposition on both patient and practitioner. The former will have neuralgia and a big bill with the physician; the latter will have the fillings to remove and replace. He takes the pulp out surgically, using local obtundents, then fills the canal for a week with antiseptics, finally filling the roots with Robinson's fibrous material, dissecting out and using long fibres, packed in one by one.

*Dr. Walker*—Close the foramen, seal the apex with what you choose—gold or lead, or whatever the case may require—and then fill the roots with gutta percha or oxy-chloride of zinc, packed in with cotton wrapped on a broach, bringing back the cotton and leaving the plastic material. Believes in preserving the nutrient functions as long as possible; would save the pulp; would cap and fill temporarily, and watch for a few days. If no trouble ensues he would then cut down the temporary filling and finish permanently. He unqualifiedly condemned wholesale devitalization because of fear of what might occur.

*Dr. Robinson*—If you will take a spring broach twice the size of a horse hair, and blunt the end upon an oil-stone, it will not go through your material. For thirty years he has taught that it is proper to extract no tooth unless it can be done with the unaided fingers. For capping exposed pulps the oxide of zinc may be mixed with carbolic acid to the consistency of cream, and then gently flowed over the exposed place. Upon this a suitable cap may be placed.

*Dr. Chisholm* — Still uses the old wood-creosote, or oil of cloves. Has capped exposed pulps that have remained alive and serviceable for fifteen years. There is only one secret in root filling—"close the door and lock it," no matter with what, so that it is done well. Has used spirits of turpentine on exposed pulps, with success.

Adjourned until 2.30 P. M.

SECOND DAY—AFTERNOON SESSION.

The subject of Operative Dentistry was continued.

*Dr. Teague*—Said there were two conditions requisite in capping pulps, an absence of pressure, and a non-conducting filling. His practice is to wash gently with carbolized water, and then flow creamy oxy-phosphate over the pulp, finishing with a good grade of amalgam. He cautions the patient against the pressure of mastication at first.

*Dr. Reese*—The surest test for impurities in the canal is permanganate of potash. When introduced into the canal, on cotton, it will turn brown if impurities remain. As to thermal changes, if the tooth is affected by cold only, there is no danger. If heat is painful there is inflammation, and the pulp is dead, or will die soon.

*Dr. McKellops*—Agrees with Dr. Morgan in cutting away dentine freely in dead teeth; otherwise the tooth will turn brown. He differs from Dr. Knapp as to opening. If the top is left intact, and the tooth filled from the side, we cannot see what is done. Neuralgia will follow filling around a calcified pulp; the branches in the roots will give excruciating pain. He can see no object in leaving a nodule, as described by Dr. Morrison. A nodule is foreign material, and should be removed. We often claim as successes what are really failures. We may not know of it, because our failures do not always come back to us; they are tired of us, and go somewhere else. In all ordinary cases of abscess he treats through the foramen, but if the canal be already filled, and there is a blind abscess, trouble cannot be avoided. He described several cases of failures, and exhibited the extracted teeth, with cotton, gold, and other materials projecting through the apex. For treatment and filling of nerve canals he uses a number 10 Swiss broach, which costs only four dollars per gross. Imbed the broach in unslacked lime, and heat to a red heat. This will make it exceedingly tough. For filling he winds such a broach with a few fibres of cotton, and carries gutta percha, dissolved in chloroform, to the apex. No man can fill a crooked nerve canal perfectly with gold, or with wire. It will either project through, or come short of the apex. First-class work cannot be done in a hurry, and therefore he uses gutta percha, with which he can take all the time he desires. When necessary he separates with waxed tape; takes a week, and thus avoids soreness, and gets plenty of room. Is always ready to give any man any amount of money to be taught how to do things in the best manner. Sometimes gold work will fail. There are cases that can be saved only with oxy-phosphate fillings, frequently renewed. Uses

Mur. Cocaine, gr. 1½.

Spts. Alcohol, dr. 1.

Chloroform, dr. 1.

for local anæsthesia, with entire success.

*Dr. C. W. Spalding*—No man will honestly advocate what he does not know he can do successfully; but no one, in the papers or in the discussion, has claimed to fill roots with gold wire, and nothing else. The apex can be closed with gold, and then it makes little difference what you fill the canal with.

*Dr. H. A. Smith*—Desired to emphasize what Dr. Harlan had said about excluding foreign elements, and, with Dr. Parmly Brown, would "make haste slowly." When a tooth is in a pathological condition, infiltrated with septic matter, every time we apply an antiseptic we are making progress towards a cure. But we must maintain this progress until the end be reached. When a healthy condition is attained, unless complete isolation from septic influences be maintained, organic matter will enter, disorganize, and the whole work must be done over again.

*Dr. Taft*—We must be guided by the condition of the dentine of each tooth. The dentine of young teeth contains much organic matter; it is not dense; septic emanations pass through readily to the periosteum. Operations successful in dense structure would not be tolerated in soft teeth. Some teeth discolor promptly; others resist for years. Different constitutions resist irritants differently. Some take up and eliminate poisons without disturbance ; others have serious periosteal trouble upon the slightest occasion. Different methods of treatment are necessary in youth and age, in health and debility. We must study the histological character of structures, and use discrimination.. The electric light gives invaluable aid in distinguishing characteristics of tooth adjuncts.

*Dr. Morgan*—Differs with Dr. Harlan as to success in removing the entire contents of the tubuli with alcohol; even though removed they will be renewed from the periosteum. We should use antiseptics to prevent decomposition and discoloration. It is impossible to save pulpless teeth in the negro; the tooth structure is too loose; there is too much organic matter; they are too like teeth of young children. To insure success in root-fillings it is necessary to drill the length of the canal with a cone-shaped bur, and you then know the length and breadth, and leave a cone-shaped opening at the apex. Then drive home a cone-shaped cylinder of gold, and you have the canal sealed. The cone-shaped bur forms the model for the cylinder. It is impossible to plug crooked roots with gold, and foretell the result. When an abscess has been treated, abnormal connective tissue is formed—eschar tissue—and this is less liable to inflammation than normal tissue. To prevent abscess, watch the case, and if soreness supervenes, apply aqua ammonia as a counter-irritant.

*Dr. J. J. R. Patrick*—The external surface of a root and the contour of the canal, will usually correspond in outline. Roots are sometimes club-shaped and contorted, but internal walls, as a rule, follow the external. I have seen a wire filling projecting beyond the apex one-half inch, and it was worn for years without trouble. As to fistulous openings, in accordance with the laws of gravitation lower teeth drain readily, and nothing is gained by waiting. Upper teeth drain through the canal; if filled, we must depend upon absorption to remove any deposits.

*Dr. Morrison*—The discussion has lasted too long already, but there are several points to which I desire to reply. Wire will not pass through the foramen if the right size is selected after trial. I doubt if Dr. Patrick will ever find a second case with wire one-half inch beyond the foramen. I will be glad to demonstrate my method, and prove its success. I am ready to give a clinic, either upon teeth in the mouth or imbedded in plaster so that I cannot see the roots, but I would prefer in the mouth. (Applause.)

*Dr. T. T. Moore*—Dr. Morrison may be able to withdraw the pulp through an aperture of one-sixteenth of an inch, but I would prefer to sacrifice a little more dentine.

On motion, the discussion was closed.

Dr. Williams read a paper, with large chart illustrations, describing his artificial crown, and the method of attachment, the latter based on mathematical and mechanical principles. The main points are a large sized but short pin of elliptical shape, conforming to that of the root (the crown consequently not liable to twist), and split at both ends to admit steel wedges, which, when driven home, expand both ends of the pin against the inside walls of the hour-glass-shaped pivot connecting the root and crown, drawing the two together and forcing out the surplus cement, which hardens while the patient bites it into perfect articulation. The wedging principle prevents all possibility of loosening; it is self-articulating, and can be inserted in one short sitting. It is apparently as permanent as the root itself. After some questions and explanations, the subject of Operative Dentistry was finally passed.

